# Enhanced anger superiority effect in generalized anxiety disorder and panic disorder

**DOI:** 10.1016/j.janxdis.2011.11.010

**Published:** 2012-03

**Authors:** Chris Ashwin, Pawel Holas, Shanna Broadhurst, Andrzej Kokoszka, George A. Georgiou, Elaine Fox

**Affiliations:** a Department of Psychology, University of Essex, Wivenhoe Park, Colchester, UK; b II Department of Psychiatry, Medical University of Warsaw, Warsaw, Poland; c Dept. of Psychology, Roehampton University, London, UK

**Keywords:** Anxiety, Threat, Generalized anxiety disorder, Panic disorder, Attention bias, Visual search

## Abstract

People are typically faster and more accurate to detect angry compared to happy faces, which is known as the anger superiority effect. Many cognitive models of anxiety suggest anxiety disorders involve attentional biases towards threat, although the nature of these biases remains unclear. The present study used a Face-in-the-Crowd task to investigate the anger superiority effect in a control group and patients diagnosed with either generalized anxiety disorder (GAD) or panic disorder (PD). The main finding was that both anxiety groups showed an enhanced anger superiority effect compared to controls, which is consistent with key theories of anxiety. Furthermore, both anxiety groups showed a differential pattern of enhanced bias towards threat depending on the crowd in the displays. The different attentional bias patterns between the GAD and PD groups may be related to the diverse symptoms in these disorders. These findings have implications for the diagnosis and treatment of anxiety.

## Highlights

► We examine anger superiority effects in controls and people with anxiety disorders. ► All participants detect anger faster and more accurately than happy faces. ► People with GAD and PD show enhanced detection of anger compared to controls. ► GAD shows different patterns of anger superiority to PD, depending on the context.

## 1 Introduction

Humans sense more information in their environment than they can effectively process, so attention is necessary to filter out unnecessary information and to focus on relevant items. Many cognitive theories of anxiety propose that biases in attention play an important role in the causation and maintenance of anxiety disorders (Beck, 1976; Eysenck, 1992; Mathews, 1990; Williams, Watts, MacLeod, & Mathews, 1988, 1997). There is now much evidence showing that high-trait anxious people and patients with clinical diagnoses of anxiety display attentional biases towards threatening information (for reviews see: Bar-Haim, Lamy, Pergamin, Bakermans-Kranenburg, & van Ijzendoorn, 2007; Mathews & MacLeod, 2005; Mogg & Bradley, 2004).

Much of the evidence for attentional biases in high anxiety has emerged from a small number of experimental paradigms, including the Stroop task, dot probe and Face-in-the-Crowd tests. In the modified Stroop task people have to name the colours of words printed in different fonts, either in a list or presented one at a time. Findings have revealed that anxious individuals show greater interference when colour-naming threatening words compared to neutral words (Williams, Mathews, & Macleod, 1996). This is proposed to reflect that attention to the negative content in distracter words interferes with performance on the central task of naming the ink colour. This effect has also been shown when words are masked to restrict awareness (MacLeod & Rutherford, 1992; Mogg, Bradley, Williams, & Mathews, 1993), and there is evidence that the Stroop effect in these modified designs also predicts the amount of later distress experienced from a disturbing life event (MacLeod & Hagan, 1992). However, it has been argued the mechanisms underlying this effect may actually reflect disruption caused by the emotional valence, rather than attentional effects (Mathews & MacLeod, 2005). In addition, some propose that word stimuli may be limited in anxiety research for a number of reasons (Bradley, Mogg, White, Groom, & de Bono, 1999; Mogg & Bradley, 2004). For example, words could represent a weaker stimulus or might have a more indirect relationship with ‘real-world’ dangers, compared to pictorial representations of threat. There is also a possible confound that anxious people may be more familiar and experienced with threat-related words compared to controls. Facial expressions of emotion might represent a more potent and ecologically valid type of stimuli for investigating biases towards threat in anxiety research.

Other experimental methods, including the dot probe and Face-in-the-Crowd paradigms, have utilised images of facial expressions to investigate attentional biases in anxiety. Dot-probe paradigms typically involve two pictures appearing briefly in a display, followed by a target probe appearing behind the location of one of the pictures. Faster responses to a target are inferred to show that attention was preferentially allocated to the picture that appeared in its location. A number of dot-probe studies have reported that people with high trait anxiety and those diagnosed with clinical anxiety show an attentional bias for threatening information, including facial expressions of emotion (Bradley et al., 1999; Mogg & Bradley, 1999, 2002; Roy et al., 2008; Waters, Mogg, Bradley, & Pine, 2008). For example, using a dot probe paradigm, Mogg and Bradley (1998) showed that people with high trait anxiety attended more to threatening faces presented at 500 ms or 1250 ms compared to a control group and people with dysphoria.

Another method used to study the detection of threat using images of emotional expressions is the Face-in-the-Crowd paradigm. Hansen and Hansen (1988) carried out the original study involving groups of photographs showing emotional expressions arranged in displays. Participants had to search the displays and decide if a discrepant face was present or not. They reported that people detected angry faces faster and more accurately than happy faces, and interpreted this to illustrate a threat superiority advantage for angry faces compared to friendly faces. However, the study was limited after discovery one of the photographs had a shadow in it, which may have affected the results (Purcell, Stewart, & Skov, 1996). Further studies developed computer-drawn schematic faces instead of photographs, to avoid visual confounds between stimulus types. These versions of the Face-in-the-Crowd task have used schematized angry, happy and neutral expression faces arranged in displays on a computer screen. Participants decide if all the faces in the display are the same, or if there is one face that is different than the rest. A number of studies with control participants have used the Face-in-the-Crowd task with schematic faces and found that angry faces are detected faster and more accurately than friendly faces, termed the anger superiority effect (Ashwin, Wheelwright, & Baron-Cohen, 2006; Eastwood, Smilek, & Merikle, 2003; Fox, Lester, Russo, Bowles, Pichler, & Dutton, 2000; Öhman, Lundqvist, & Esteves, 2001). These findings support ideas of an evolutionarily developed threat detection module that preferentially detects stimuli in the environment that signal threat and allocates attentional resources towards them (Öhman, 1986; Öhman & Mineka, 2001; Öhman et al., 2001).

To date only a small number of studies have been reported using the Face-in-the-Crowd task with people who are high anxiety or diagnosed with anxiety disorders. Most of these studies have included people with non-clinical high anxiety or with diagnoses specific to social anxiety. Byrne and Eysenck (1995) used this paradigm with photographs of people expressing angry and happy expressions in displays and found that individuals high in subclinical trait anxiety were faster in detecting angry faces compared to low trait-anxiety. Gilboa-Schechtman, Foa, and Amir (1999) used a similar Face-in-the-Crowd design to Byrne and Eysenck with photographs and reported that people with social anxiety disorder showed greater attentional biases for angry versus happy faces compared to controls. A study by Juth, Lundqvist, Karlsson, and Öhman (2005) utilized the Face-in-the-Crowd paradigm with schematic faces, instead of photographs, and found that people with high social anxiety showed more effective detection of angry compared to happy faces. Another Face-in-the-Crowd study using schematic faces and different display sizes found that people with social anxiety disorder had shallower slopes for detecting angry faces compared to happy faces (Eastwood et al., 2005), illustrating that angry faces capture attention more easily than happy faces in those with social anxiety. They further reported that people diagnosed with PD also had a greater attentional bias towards angry faces, comparable with the social anxiety group. A meta-analysis by Bar-Haim et al. (2007) looked at 172 different studies measuring bias towards threatening information in people with and without high anxiety using a variety of experimental paradigms. The authors reported a robust threat-related bias with a low to medium effect size was evident in people who are diagnosed with anxiety disorders or have high sub-clinical measures of trait anxiety.

Generalized anxiety disorder (GAD) and panic disorder (PD) are both clinically diagnosed anxiety disorders. GAD is characterized by heightened anxiety and tension alongside difficulty in relaxing. People with GAD have excessive and often irrational worry about aspects of everyday life (APA, 1994). Their worry is characterized by repeated negative thoughts about possible threat, which may emerge from attempts at avoidance or coping (Borkovec & Roemer, 1995). This pervasive worry is beyond that normally experienced in everyday life, and expresses as chronic and exaggerated anxiety. Therefore, individuals with GAD tend to always be anticipating disaster and worrying about issues such as health, money, family, friends, and work. In contrast to the chronic low-level anxiety found in GAD, panic disorder (PD) involves unexpected episodes of intense fear accompanied by physical symptoms, and persistent apprehension over their recurrence or consequences (APA, 1994). A strong component of PD is the fear of embarrassment or humiliation from others. In fact, individuals often report a desire to avoid or escape public places because they would feel embarrassed or humiliated if they had a panic attack there. Since social elements are an important factor, it is thought people with PD might be biased towards cues of social evaluation, such as emotional expressions (Eastwood et al., 2005).

While GAD and PD present with dissimilar behavioural profiles, the nature of any differences between PD and GAD in their attentional biases towards threat is currently unknown. One idea is there might be a common core threat-related attention bias shared by the various anxiety disorders, with attentional differences between disorders emerging from other factors (Bar-Haim et al., 2007). Alternatively, distinctive patterns of attentional biases related to behavioural symptoms might be evident across various anxiety disorders. While there is some initial evidence to suggest various anxiety disorders might show differences in how they process threatening information, there is a lack of experimental findings in this area (Amir et al., 1996; Vrana, Roodman, & Beckham, 1995). Research has shown differences between anxiety disorders in the timing of attentional biases, with some disorders showing early biases towards threat and later biases away from threat (Mogg & Bradley, 2004, 2006). However, Eastwood et al. (2005) reported that social phobics and those with PD had similarly enhanced attention towards threatening faces. There have been a number of mixed findings to date, so further research is needed to better understand the nature of cognitive biases in anxiety disorders.

### 1.1 Experimental aims

The main aim of the present study was to investigate attentional biases towards angry faces in control participants and clinical patients diagnosed with either GAD or PD using the Face-in-the-Crowd task with schematic faces. Schematic faces were used because they are highly matched on low-level visual characteristics, which minimizes potential visual confounds that can occur between expressions in real photographs (Fox et al., 2000; Öhman et al., 2001). We predicted that the GAD and PD would show enhanced anger superiority effects compared to controls. Since PD is associated with heightened concerns about socially observed anxiety, we hypothesised people with PD might show even greater anger superiority effects compared to those with GAD, especially when crowds contained emotional faces.

## 2 Materials and methods

### 2.1 Participants

The study was conducted in the Laboratory of Sexology and Psychotherapy, at the II Department of Psychiatry, Medical University of Warsaw. 66 volunteers were recruited to take part in the study. Participants included 18 clinical patients (13 females) who had received a diagnosis of GAD, and 17 clinical patients (15 females) who had received a diagnosis of PD by trained psychiatrists at the outpatient clinic. Patients were initially referred to the clinic by a psychiatrist based on a diagnosis of anxiety disorders according to international criteria (WHO, 1992). Diagnosis of the patients was then confirmed at the clinic using either the official Polish version of the Mini-International Neuropsychiatric Interview (MINI) version 5.0.0 (Masiak & Przychoda-Masiak, 2002; Sheehan et al., 1997) or further international criteria (APA, 1994). The selection criterion for the patient groups in the study was a primary diagnosis of GAD or PD, with no evidence of psychosis or organic brain damage. Consensual diagnosis by members of the clinical team was a requirement for selection to the research. In addition, 31 people with no history of psychiatric disorders (19 females) were recruited from the community to serve as controls. All participants completed questionnaire measures of state and trait anxiety, depression, worry and attentional control. All participants had normal or corrected-to-normal vision, and gave written informed consent to take part.

### 2.2 Materials

The Face-in-the-Crowd task was run on Macintosh Power PC G3 with a 15-in. LCD monitor for display. The presentation of the experiment and responses of the participants were controlled by PsyScope (Cohen, MacWhinney, Flatt, & Provost, 1993). The face stimuli were schematic faces used with permission from previously published research (Öhman et al., 2001). Each of the displays contained four schematic faces arranged in a 2 × 2 matrix. Each of the targets appeared equally in all four locations. Each face took up 3.9° × 3.9° of visual angle. The closest distance from the center of the face to the central fixation point was 6.7° of visual angle and the closest distance from the center of one face to another face was 7.6° of visual angle.

#### 2.2.1 Questionnaire measures

Five self-report questionnaires were completed before participants began the computerized tasks. The Spielberger State-Trait Anxiety Inventory (STAI: Spielberger, Gorsuch, Lushene, Vagg, & Jacobs, 1983) is a 40-item self-report measure of anxiety. The first 20 items (STAI-S) measure state anxiety, or how the subject feels right now. The second 20 items (STAI-T) assess trait anxiety, or how the subject generally feels. Each item is rated using a scale from 0 (not at all) to 3 (very much so). The range of scores on both measures varies from 0 to 60, with higher scores indicating greater anxiety. A number of studies have shown reliable psychometric properties for the STAI (e.g. Spielberger et al., 1983). The Polish version (Parnowski & Jernajczyk, 1977) of the Beck Depression Inventory (BDI: Beck, Ward, Mendelson, Mock, & Erbaugh, 1961) was used to assess the severity of depression. The BDI is a 21 item questionnaire composed of items relating to depression symptoms, to measure the severity of depression. It has a four-point scale for each item ranging from 0 to 3, with scores ranging from 0 to 63. Higher scores represent more severe symptoms of depression. It has good internal consistency, with a Cronbach's alpha coefficient in adults reported to be around 0.85 (e.g. Ambrosini, Metz, Bianchi, Rabinovich, & Undie, 1991). The Beck Anxiety Inventory (BAI: Beck & Steer, 1990) is a 21 item questionnaire measuring the physiological and cognitive aspects of anxiety. It consists of descriptive statements of anxiety symptoms which are rated on a 4-point scale, from 0 (Not at all) to 3 (Severely; I could barely stand it). The total score on the BAI ranges from 0 and 63, and it has good internal consistency with Cronbach's alpha reported of .92–.94 for adults (Beck & Steer, 1990). Finally, the short version of the Attentional Control Scale (ACS: Derryberry & Reed, 2002) was included, which is a 20 item questionnaire measuring the degree of attentional control of participants. Items are scored on a 4-point scale from 0 (Never) to 3 (Always), with the total score ranging from 0 to 60. Higher scores reflect greater levels of attentional control. Studies have shown good internal reliability of the ACS in adults, reporting Cronbach's alpha of .87 (Muris, Meesters, & Rompelberg, 2007).

### 2.3 Design and procedure

Participants first completed the questionnaires at a desk in a quiet room, followed by the experimental task. Each trial of the visual search task began with the presentation of a crosshair for 500 ms, which was followed by one of the displays containing four faces for 300 ms. This display time was chosen to limit the number of saccades participants could make while viewing the displays. Participants pressed a button on the response box to indicate whether all the faces in the display were the same, or whether there was one that was different from the rest. A trial ended when the participant responded or after 2000 ms, then it moved on to start the next trial after a 1000 ms blank screen ITI.

There were 96 target-present trials, where a target appeared in a crowd of distracter faces. In half the trials, the angry and happy face targets appeared in crowds of emotional faces, i.e. angry targets in happy crowds and happy targets in angry crowds. In the other half of the trials, the angry and happy face targets appeared in crowds of neutral faces. There were 24 trials of each target–distracter combination type, creating the total of 96 target-present trials. There were also 96 target-absent trials where all the faces were the same type (i.e. angry, happy or neutral), with 32 trials of each display type. This resulted in a total of 192 trials for the experiment. The order of presentation for the different trial types was randomized throughout the experiment. Therefore, participants did not know before the start of each trial which type of target–distracter combination was going to appear.

### 2.4 Statistical design

The various group characteristics were compared using independent-sample *t*-tests, and are reported in Table 1. The primary measures of interest were mean response latencies and mean accuracy scores, and for the target-present trials these were analysed using a repeated measures general linear model (GLM) ANOVA with Target (Angry vs. Happy) and Distracter (Neutral vs. Emotional) as the within-subject factors and Group (Controls vs. GAD vs. PD) as the between-subject factor. Mean response latencies and mean accuracy scores for target-absent displays were analysed using a GLM ANOVA having Valence (Angry vs. Happy vs. Neutral) as the within subjects factor and Group (Controls vs. GAD vs. PD) as the between-subjects factor.

## 3 Results

The groups did not differ in terms of age or sex, however the psychiatric groups differed from the controls on measures of anxiety and depression as expected (see Table 1).

### 3.1 Target-present displays

The measures of interest were mean response latency and accuracy scores, which were both analysed using a repeated measures general linear model (GLM) ANOVA with Target (Angry vs. Happy) and Distracter (Neutral vs. Emotional) as the within-subject factors and Group (Controls vs. GAD vs. PD) as the between-subject factor.

#### 3.1.1 Response latencies

Results revealed there was no main effect of Group, *F*(2,63) = 2.07, *ns*, $\eta_{p}^{2}=.10$, showing that all groups were performing the same overall for the experiment. However there was a main effect of Target, *F*(1,63) = 74.12, *p* < .001, $\eta_{p}^{2}=.34$, with angry targets (942.6 ms) being detected faster than friendly targets (1034.0 ms). There was also a main effect of Distracter, *F*(1,63) = 39.42, *p* < .001, $\eta_{p}^{2}=.53$, with quicker response times when distracters were neutral (967.1.0 ms) compared to when they were emotional (1009.5 ms).

**Fig. 1:**
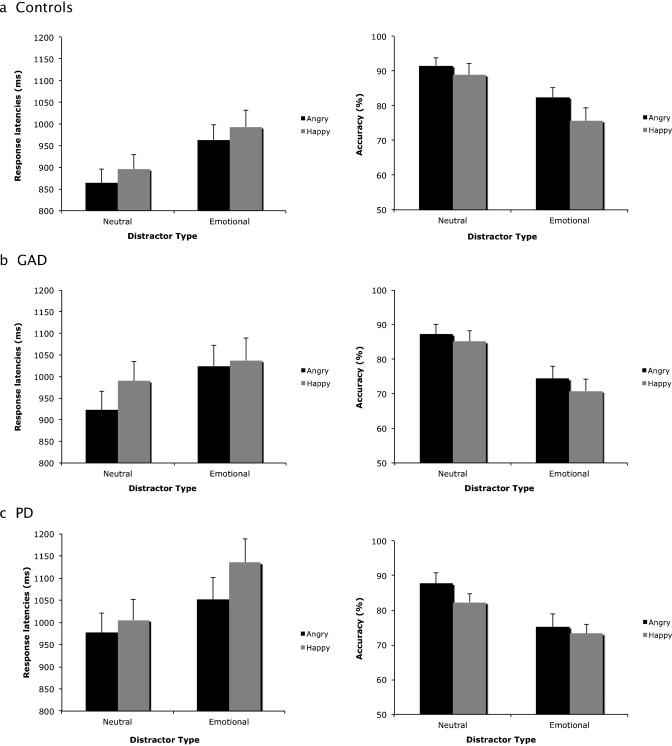
Response latencies (left side) and accuracy scores (right side) in the target-present trials to detect angry and happy faces across emotional and neutral distracters for the Control group (a), GAD group (b), and PD group (c).

Importantly, there was a three-way interaction between Target, Distracter and Group, *F*(1,63) = 6.25, *p* < .01, $\eta_{p}^{2}=.21$. To interrogate this interaction further, and to test our main hypothesis about an enhanced anger superiority effect in the anxiety groups, we first calculated ‘difference scores’ for each group by subtracting the RTs to angry targets from RTs to happy across both neutral and emotional distracter conditions. We then carried out independent sample *t*-tests on the difference scores between the anxiety groups and the controls to test our main hypothesis about an enhanced anger superiority effect in anxiety. Results showed that the difference score for the GAD group was significantly greater compared to controls when crowds were neutral, *t*(47) = 2.09, *p* < .05, *d* = .63, but not when crowds were emotional, *t*(47) = 0.74, *ns*, *d* = .23. The PD group had a significantly greater difference score compared to controls when crowds were emotional, *t*(46) = 2.01, *p* < .05, *d* = .65, but not when crowds were neutral, *t*(46) = 0.23, *ns*, *d* = .07 (see Fig. 1).

We then ran independent sample *t*-tests between the two anxiety groups to test our third aim about differences in attentional bias between anxiety disorders. The GAD group showed a greater difference score than the PD group when crowds were neutral, *t*(33) = 2.07, *p* < .05, *d* = .70, while the PD group showed a greater difference score than the GAD group with emotional crowds, *t*(33) = 3.17, *p* < .01, *d* = 1.07 (see Fig. 1).

#### 3.1.2 Accuracy data

The accuracy scores were analysed using an ANOVA with Target (Angry vs. Happy) and Distracter (Neutral vs. Emotional) as the within-subject factors and Group (GAD vs. PD vs. Controls) as the between-subject factor. There was a significant main effect of Target *F*(1, 63) = 75.97, *p* < .001, $\eta_{p}^{2}=.16$, with angry faces (87.2%) being detected more accurately than happy faces (75.3%) (see Fig. 1). There was also a significant main effect of distracter *F*(1, 63) = 17.05, *p* < .001, $\eta_{p}^{2}=.52$, showing that people were more accurate when displays had neutral (83.1%) compared to emotional distracters (79.4%) (see Fig. 1). There were no significant main effects or interactions involving Group (all *p* > .05).

### 3.2 Target-absent displays

#### 3.2.1 Response latencies

The measures of interest were mean response latency and accuracy scores, which were both analysed using a GLM ANOVA with Valence (Angry vs. Happy vs. Neutral) as the within-subject factor and Group (GAD vs. PD vs. Controls) as the between-subject factor.

**Fig. 2:**
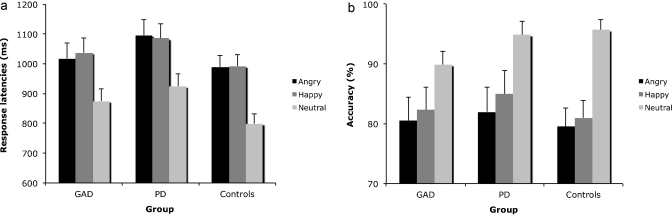
Response latencies (a) and accuracy scores (b) in the target-absent trials to detect all-angry, all-happy, and all neutral displays for the Control, GAD, and PD groups.

Results with response latencies showed there was a significant main effect of Valence *F*(2,62) = 92.66, *p* < .001, $\eta_{p}^{2}=.76$. Post hoc pairwise comparisons showed that response times for all-neutral displays (866.42 ms) were less than both the all-angry displays (1034.54 ms), and the all-happy displays (1038.54 ms), while there was no difference between the all-angry and the all-happy displays (see Fig. 2a). There was no main effect or interaction involving Group (all *p* > .05).

#### 3.2.2 Accuracy data

Results revealed a significant main effect of Valence, *F*(2,62) = 26.14, *p* < .001, $\eta_{p}^{2}=.48$ (see Fig. 2b). Post hoc pairwise comparisons showed that participants were more accurate for the all-neutral displays (93.5%) compared to both the all-angry displays (80.7%), and the all-happy displays (82.7%). There was no difference in accuracy between the all-angry and the all-happy displays. There was no significant main effect or interaction with Group (all *p* > .05).

## 4 Discussion

The present study revealed that all three groups showed the typical anger superiority effect, with faster and more accurate detection of angry faces versus happy faces. This is consistent with the idea of an evolutionarily developed threat evaluation system that preferentially detects stimuli in the environment that signal threat (Öhman, 1986; Öhman & Mineka, 2001; Öhman et al., 2001). Responding rapidly and successfully to threat is critical for survival, so it is advantageous for threat-related information to be processed in a highly efficient manner compared to other types of information. According to Öhman and colleagues (e.g. Öhman, 2002; Öhman & Mineka, 2001), there is an evolutionarily shaped fear module sub served by neural circuits centred on the amygdala, which detects and coordinates responses to threatening information, such as angry faces. Perceiving threatening stimuli interrupts ongoing cognition and this information becomes prioritized, which then provides additional processing of the threat.

In healthy individuals, a normal functioning threat detection system would be adaptive for survival. However, differences in sensitivity or threshold of this threat-detection module are thought to produce enhanced threat processing biases in anxiety disorders. The main clinical finding of interest in the present study was that both the GAD and PD groups showed an enhanced anger superiority effect compared to the controls. This result is in line with cognitive models of anxiety proposing a greater bias towards threatening information in the development and maintenance of anxiety disorders (Williams et al., 1988, 1997), and a number of previous studies reporting attentional biases towards threatening faces in highly anxious people using various paradigms (Bradley et al., 1999; Gilboa-Schechtman et al., 1999; Mogg & Bradley, 1999, 2002; Roy et al., 2008; Waters et al., 2008). A meta-analysis by Bar-Haim et al. (2007) including 172 different empirical studies found there are consistent and reliable findings of a threat-related bias in anxiety. For example, Eastwood et al. (2005) used a similar Face-in-the-Crowd paradigm to the present study and found enhanced detection of threatening faces in people with PD and social phobia. The results presented here replicate this previous finding of an attentional bias towards anger in PD, and extend the findings to also include GAD. The present findings suggest the amygdala-based fear module may be hypersensitive in those with high anxiety (Öhman, 2007). Indeed neuroimaging experiments have shown early and enhanced activation of the amygdala to negative information in people with anxiety disorders compared to controls (Evans, Wright, Wedig, Gold, Pollack, & Rauch, 2008; Larson, Schaefer, Siegle, Jackson, Anderle, & Davidson, 2006; Schienle, Schafer, Walter, Stark, & Vaitl, 2005; Stein, Goldin, Sareen, Zorrilla, & Brown, 2002).

The second main clinical finding of interest was that the two anxiety groups in the present study showed different patterns of enhanced bias towards anger, depending on the type of faces in the crowds. The PD group had a greater anger superiority effect compared to the other groups when targets appeared in crowds with emotional faces, consistent with the hypotheses. However, the GAD group showed enhanced anger superiority versus the other groups when the targets appeared within crowds of neutral faces. This shows that the social context is important for determining the nature and type of bias for different anxiety disorders. Measures taken of anxiety and depression during the experimental session did not differ between the anxiety groups. Therefore, the distinct patterns of attentional biases are likely to relate to other important factors rather than general anxiety states. These not only include the social context of the stimuli used in the experiment, but also likely involve distinctions in the behavioural characteristics and social-evaluative fears between these disorders. While attentional biases are common to all anxiety disorders, the precise content of the bias tends to be related to unique features that are significant to specific disorders (Craske & Waters, 2005; Williams et al., 1988, 1997).

The enhanced threat detection by the GAD group occurred while searching through crowds of neutral distracter faces for angry target faces, with no facilitation of threat detection when crowds contained happy distracter faces. This is consistent with ideas that biases are most evident for GAD when comparing emotional and neutral items within the same display (Mogg & Bradley, 1998, 1999). GAD is characterized by chronic and exaggerated worry related to everyday events, such as work/school performance, health, money, family or friends. The anxiety-related symptoms in GAD are less intense compared to other anxiety disorders such as PD, where people experience panic attacks involving extreme fear (Noyes & Hoehn-Saric, 1998). In everyday life intense emotional events where crowds of people with emotional expressions are all looking towards you are not commonplace events. Instead, people with GAD may be more attuned to perceiving threat in contexts more typical of everyday life with less emotional arousal or social stress. In the present study, searching for an angry face within a neutral crowd may be more related to the chronic worry about everyday life events which personifies GAD. Furthermore, there was not an enhanced bias for GAD when searching for angry targets amongst emotional distracters, which may be due to them perceiving happy faces within these crowds. Seeing happy faces may actually serve to alleviate worries and help reduce any enhanced threat detection in people with GAD, and may account for why the enhancement of threat detection in GAD was only seen within the neutral distracter crowds. Therefore, the social-evaluative fears in people with GAD may be more tuned to seeing threat within neutral-related contexts, more akin to everyday life events.

The opposite pattern of bias findings was seen in the PD group. They showed enhanced threat detection while searching for angry targets within crowds of happy faces, but no enhancement when searching neutral crowds. PD involves sudden and intense anxiety states more closely linked to bodily sensations and their contexts, and the fear of evaluation about these bodily sensations. The social-evaluative fears in this disorder may be more associated with crowds featuring all emotional faces looking towards them, as these have higher emotional intensity and greater potential signs of social evaluation compared to neutral crowd displays. On the other hand, the reduced emotional intensity in the displays containing neutral face crowds may actually help to alleviate anxiety to some degree in those with PD, as these contain fewer emotional expressions and, therefore, potential signs of social evaluation. Panic attacks are usually related to trigger factors which are social-emotional in nature, while the increase of anxiety in GAD is more likely to occur in situations of decreased stimulation, passivity, and decreases in goal-oriented activity. There is some evidence to support this idea from a study looking at physiological reactivity in people with GAD versus PD in the form of respiration measures (Wilhelm, Trabert, & Roth, 2001). They found the two disorders could be differentiated from each other based on their physiological behaviour, in terms of respiration in response to emotional stimuli. More specifically, the PD group showed greater physiological reactivity compared to GAD. This provides a ‘bottom-up’ interpretation of the disparity in attentional biases between GAD versus PD, involving differences in physiological reactivity that may be influenced by the affective intensity of stimuli.

Another possible explanation for the bias differences is a more ‘top-down’ influence of attentional control. All the groups in the present study differed from each other on their ACS scores, with the control group scoring the lowest compared to the two anxiety groups, and the GAD group having higher attentional control than the PD group. The ACS is proposed to measure top-down mechanisms controlling attentional resources, with higher attentional control relating to voluntary coping strategies when processing information perceived as threatening (Derryberry & Reed, 2002). Attentional control is thought to help regulate emotional responses and anxiety. Therefore, these findings may suggest differences between the anxiety groups in higher level voluntary control of the attentional processing of threat within different contexts. Interestingly, cognitive-behavioral therapy (CBT) of GAD focuses on restructuring dysfunctional beliefs and meta-beliefs, while CBT of PD focuses more on the learning of relaxation, the recognition of trigger factors, and the mechanisms underlying the exaggeration of threatening experiences. Therefore, the CBT for GAD focuses on mental processes, while for PD it focuses on emotional reactions related with bodily experiences and their social consequences (Wells, 1997).

Our findings cannot be accounted for by a speed-accuracy trade-off in responding to angry faces, where people might be faster but also making more errors. Instead, participants in the study were not only faster in responding to angry compared to happy faces, but they were also more accurate in their detection of angry faces. This runs counter to the idea of a speed-accuracy trade-off. Another possible explanation for the results is that people in the anxiety groups were not actually faster in detecting the angry face targets, but instead might have been faster in scanning through the distracter crowds of happy expressions when searching for angry face targets within displays. However, differences between the groups in scanning the distracter crowds does not help explain the results, as no group differences in response latencies or accuracy were found for responding to crowds of happy versus angry faces when no targets were present. This suggests they were equally fast in scanning through both happy and angry crowds when detecting the targets. The present findings also cannot be explained by differences in detecting low-level visual characteristics in the faces, such as curved or angled lines in the stimuli (Treisman, 1980). The angry and happy schematic faces were very highly matched to each other on all visual characteristics, which was why they were chosen as stimuli. The only difference between the angry and happy faces was the orientation of key features which produced the corresponding emotional expressions. Therefore, we feel the best explanation for the present findings is an enhanced attentional bias towards threatening information in clinical anxiety, consistent with previous experimental results and key theories.

The present findings have potential implications for the assessment and treatment of anxiety disorders. Diagnostic differentiation between different anxiety disorders can be difficult when it is based solely on clinical interviews. Therefore, experimental paradigms such as the Face-in-the-Crowd task could be used to help aid in more accurate diagnosis by objectively revealing abnormal biases towards threatening information in patients, as well as the nature of the bias. Running the experimental task before and after treatment could also help to reveal the effectiveness of interventions, for example by showing whether treatment has been successful in reducing the bias towards threat. Of course this application would require high reliability and specificity of the task to consistently show enhanced biases in people with anxiety, and also for revealing different patterns of biases between various anxiety disorders. The present study generally had medium effect sizes for differences between groups; however more research is needed to test the reliability and consistency of findings across many clinical studies. Similar experimental paradigms could also potentially be used to help reduce enhanced attentional biases in those with high anxiety. Fox, Ashwin, Zougkou, and Broadhurst (submitted) recently used a dot probe paradigm with people who were high or low in spider phobia, and manipulated the location of the target to always be away from spider pictures for those high in spider fear. They found that after many experimental trials there was a reduced attentional bias towards spiders in those with high spider phobia, and the reduction in bias was also accompanied by less aversive ratings of spider-related photos. There is growing evidence that such attentional training, or cognitive bias modification procedures, can alleviate anxious symptomatology in a variety of anxiety disorders (for reviews see Browning, Holmes, & Harmer, 2010; Hakamata et al., 2010; MacLeod, Koster & Fox, 2009), including GAD (Amir, Beard, Burns, & Bomyea, 2009) These potential clinical applications of cognitive experimental paradigms are important because although CBT treatments are reported to be effective for many people diagnosed with anxiety disorders (Butler, Chapman, Forman, & Beck, 2006), as many as one third of patients do not receive significant treatment benefits and few patients actually achieve full recovery (Ballenger, 1999).

While the simplicity of the experimental paradigm makes it well-suited for psychiatric research, one limitation of the present study is that the display sizes were always the same throughout. This was due to time constraints with the availability of the anxiety participants during testing sessions. Having different display sizes would have allowed for the analysis of detection slopes for the angry and happy targets across the different sizes for each group (Eastwood et al., 2005; Frischen, Eastwood, & Smilek, 2008). This additional factor would have helped to reveal more specificity about whether pre or post attentional mechanisms were involved in the present findings. Therefore, at present we cannot determine whether enhanced effects by GAD and PD groups were due to better awareness of the emotional targets or due to response biases once the targets were detected. We have been cautious in our interpretations regarding the biases involved and not speculated about whether the effects involved pre or post attentive processes. Further studies of this type in anxiety disorders should include different size displays, to help determine more specifically the type of attentional mechanisms involved. Further research should also include greater numbers of clinical patients, as the participant numbers in the present study were limited.

## Figures and Tables

**Table 1:** Mean demographic and questionnaire measures for the GAD, PD, and Control groups (standard deviations in parentheses).

	Group			
	GAD (*n* = 18)	PD (*n* = 17)	Controls (*n* = 31)	Statistics
Age	32.06 (10.2)	32.76 (9.22)	29.26 (9.20)	*F* = .922, *ns*
Sex ratio m:f	5:13	2:15	12:19	*X*^2^ = 3.9, *ns*
STAI-T	55.47^a^ (9.23)	52.0^b^ (9.30)	41.29 (9.18)	*F* = 15.4, *p* < .001
STAI-S	50.08^a^ (8.56)	44.41^b^ (10.8)	34.81 (10.9)	*F* = 13.5, *p* < .001
BDI	17.89^a^ (7.76)	16.35^b^ (9.06)	7.23 (5.94)	*F* = 15.2, *p* < .001
BAI	22.01^a^ (11.5)	23.18^b^ (13.7)	7.65 (8.71)	*F* = 15.4, *p* < .001
ACS	56.0^a^^,^^c^ (8.93)	50.18 (7.21)	45.52 (11.0)	*F* = 6.58, *p* < .01

*Note*: STAI-T = Trait scale of the State-Trait anxiety inventory; STAI-S = State scale of the State-Trait anxiety inventory; BDI = Beck Depression Inventory; BAI = Beck Anxiety Inventory; ACS = Attention Control Scale.

a GAD > Controls (*p* < .01).

b PD > Controls (*p* < .01).

c GAD > PD (*p* > .05).
